# Excimer versus Femtosecond Laser Assisted Penetrating Keratoplasty in Keratoconus and Fuchs Dystrophy: Intraoperative Pitfalls

**DOI:** 10.1155/2015/645830

**Published:** 2015-09-21

**Authors:** Moatasem El-Husseiny, Berthold Seitz, Achim Langenbucher, Elena Akhmedova, Nora Szentmary, Tobias Hager, Themistoklis Tsintarakis, Edgar Janunts

**Affiliations:** ^1^Department of Ophthalmology, Saarland University Medical Center UKS, Homburg/Saar, Germany; ^2^Experimental Ophthalmology, University of Saarland, Homburg/Saar, Germany

## Abstract

*Purpose*. To assess the intraoperative results comparing two non-mechanical laser assisted penetrating keratoplasty approaches in keratoconus and Fuchs dystrophy. *Patients and Methods*. 68 patients (age 18 to 87 years) with keratoconus or Fuchs dystrophy were randomly distributed to 4 groups. 35 eyes with keratoconus and 33 eyes with Fuchs dystrophy were treated with either excimer laser ([Exc] groups I and II) or femtosecond laser-assisted ([FLAK] groups III and IV) penetrating keratoplasty. Main intraoperative outcome measures included intraoperative decentration, need for additional interrupted sutures, alignment of orientation markers, and intraocular positive pressure (vis a tergo). *Results*. Intraoperative recipient decentration occurred in 4 eyes of groups III/IV but in none of groups I/II. Additional interrupted sutures were not necessary in groups I/II but in 5 eyes of groups III/IV. Orientation markers were all aligned in groups I/II but were partly misaligned in 8 eyes of groups III/IV. Intraocular positive pressure grade was recognized in 12 eyes of groups I/II and in 19 eyes of groups III/IV. In particular, in group III, severe vis a tergo occurred in 8 eyes. *Conclusions*. Intraoperative decentration, misalignment of the donor in the recipient bed, and need for additional interrupted sutures as well as high percentage of severe intraocular positive pressure were predominantly present in the femtosecond laser in keratoconus eyes.

## 1. Introduction

Keratoconus (KC) and Fuchs' dystrophy (Fuchs) are the leading indications for penetrating keratoplasty (PKP) [1]. The cornea surgeon's main attention in corneal transplantation has shifted from preserving a “clear graft” to achieving a good refractive outcome.

**Figure 1:**
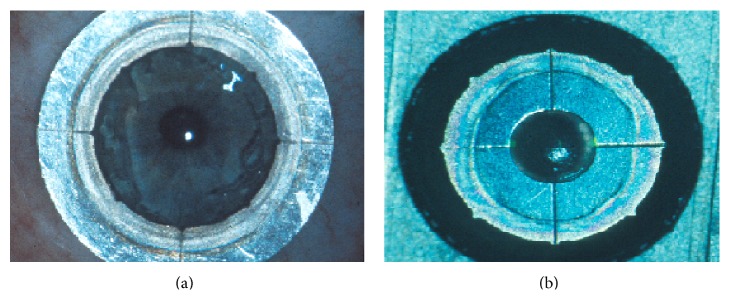
Left: 8 “orientation notches” at the recipient mask lying on a patient's cornea. Right: 8 “orientation teeth” at the donor mask lying on a corneoscleral button in an artificial anterior chamber.

The nonmechanical excimer laser trephination (Exc) has been first introduced in 1989 at the University Eye Hospital of Erlangen (Germany) [1–3]. It has been frequently reported that this technique yielded a better refractive outcome in comparison to manual trephination, particularly lower postoperative keratometric astigmatism, higher regularity of topography, and improved visual acuity [4, 5]. It ensures an outstanding perpendicular incision profile. Such cut edges in combination with “orientation teeth” (Figure 1) potentially reduce “vertical tilt” and “horizontal torsion” of the graft in the recipient bed, thus improving the visual performance after transplantation [5].

However, despite the promising results, the Exc PKP did not get widely spread, because corneal surgeons did not have an excimer laser in their operating theater. Instead, newer technologies were introduced, particularly femtosecond laser-assisted keratoplasty (FLAK), which got spread more widely since 2006 [6–8]. Historically, the femtosecond laser has been mainly used in refractive surgery, for example, for flap preparation in LASIK, intracorneal ring segment implantation in keratoconus patients, or antiastigmatic incisions following PKP [9–11].

This new technique allowed creating reproducible, customized trephination patterns. The most common trephination profiles are the “mushroom” and the “top hat” profile [12] as well as the more complex “zig-zag” profile [13]. These shaped wound configurations offer the advantages of better donor-recipient fit and increased donor-host junction surface area contact, both resulting in faster wound healing and earlier suture removal, thus potentially promoting rapid visual recovery [14]. In addition, in vivo confocal microscopy (IVCM) after FLAK showed earlier regrowth of corneal nerves in both the peripheral and central stroma compared to conventional PKP [15].

To the best of our knowledge, this is the first study to compare two nonmechanical (excimer and femtosecond laser) laser-assisted PKP in keratoconus and Fuchs dystrophy. The purpose of this work is to demonstrate the intraoperative results regarding the centration of the graft, necessity of completion of graft and donor incisions with scissors, anterior stepping, gaping, necessity of additional interrupted sutures, positive pressure during the surgery (vis a tergo), and the proper alignment of the orientation markers in host and graft.

## 2. Patients and Methods

In this prospective randomized clinical single-center study, 68 patients (age 18 to 87 years) with keratoconus or Fuchs dystrophy (phakic or pseudophakic eyes that underwent a primary central PKP) were randomly distributed to 4 groups: 35 eyes with keratoconus and 33 eyes with Fuchs dystrophy were treated either with excimer laser ([Exc] groups I and II) and with femtosecond laser-assisted ([FLAK] groups III and IV) penetrating keratoplasty. Exclusion criteria were repeated PKP and simultaneous cataract surgery because during the triple procedure, the iris-lens diaphragm is not stable and, therefore, might influence the main outcome measures of this study. All patients agreed to the informed consent. The study was approved from the Ethics Committee of the Saarland University, Germany.

All surgical procedures were carried out by one surgeon (BS) and under general anesthesia. In the study, a 193 nm excimer laser (MEL 70, Carl Zeiss Meditec, Jena, Germany) with a 35 Hz repetition rate and spot size of 1.2 mm, in combination with conventional donor/recipient masks, and the 60 KHz IntraLase FS Laser [AMO (Abbott Medical Optics), Abbott Park, IL, USA] have been used.

### 2.1. Indications

Exc and FLAK were performed in patients with keratoconus and with Fuchs' endothelial dystrophy (if they presented advanced stages of the disease including scarring, thus they were not suitable for lamellar techniques such as DSAEK or DMEK).

### 2.2. Main Outcome Measures

Main intraoperative outcome measures included ultrasound pachymetry AL-3000 (Tomey, Nagoya, Japan) at the center and in 4 midperipheral points at 0°, 90°, 180°, and 270° of the donor, complications of laser trephination, trephination time, anterior gaping, graft override, need for additional interrupted sutures to achieve proper donor-host alignment, and alignment of orientation markers and positive vitreous pressure (vis a tergo) depicted in three grades (0 = no intraoperative pressure, 1 = iris prolapse till the level of the corneal incision, and 2 = iris prolapse beyond the level of corneal incision). Graft decentration was measured at the end of the operation by measuring the distance between limbus and graft with calipers at the 12- and 6-o'clock position. Decentration was considered if the difference between the two distances was more than 0.5 mm. A measurement prior to incision was not possible because of the suction ring for the femtolaser which prevents view on the limbus area.

### 2.3. Excimer Laser Trephination

**Figure 2:**
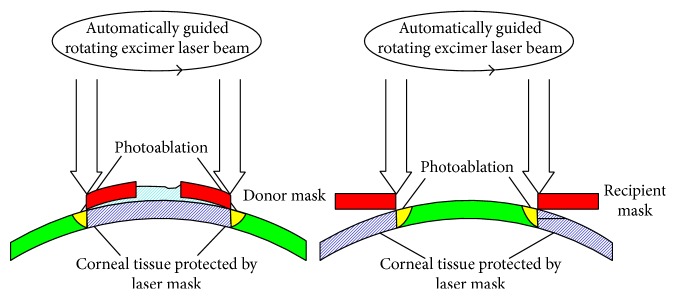
Donor and recipient trephination through rotating excimer laser beam.

Trephination was performed using the 193 nm excimer laser along metal masks with eight orientation teeth/notches. Mean patient age in keratoconus was 37.4 ± 14.2 and in Fuchs 69.8 ± 8.9 years. For donor trephination from the epithelial side using the 193 nm excimer laser MEL 70, a circular metal aperture mask (diameter: 8.1 mm; central opening: 3.0 mm for centration; thickness: 0.5 mm; weight: 0.173 g; eight orientation teeth: 0.15 × 0.3 mm, Figure 1) was positioned on a corneoscleral button (16 mm diameter) fixed in an artificial anterior chamber under microscopic control (Figure 2). After perforation, the remaining stromal lamellae and Descemet's membrane were cut with curved corneal microscissors. The donor oversize was 0.1 mm in all cases.

For recipient trephination, a corresponding circular metal mask was used (diameter: 12.5 mm; central opening: 8.0 mm; thickness: 0.5 mm; weight: 0.29 g; eight orientation notches: 0.15 × 0.3 mm, Figures 1 and 2). The mask holds without additional stabilization because of the horizontal orientation of the patient's head. The laser beam is guided automatically along the edge of the mask without ablating the central cornea. After focal corneal perforation, the remaining deep stromal lamellae and Descemet's membrane were cut with curved corneal microscissors.

### 2.4. Femtosecond Laser Trephination and Profiles

Mean patient age in keratoconus group was 40.2 ± 14.0 years and in the Fuchs group it was 69.2 ± 12.0 years. In all cases, we used energy of 0.1 *μ*J less than the maximum energy in the posterior side cut, 0.5 *μ*J less than the maximum energy in the anterior side cut, and 0.4 *μ*J less than the maximum energy in the ring lamellar cut (2.3 to 2.9 *μ*J). The 8 alignment incisions in both the donor and recipient were created as follows: energy of 1.5 *μ*J, length of 1000 *μ*m, width of 50 *μ*m, spot separation of 6 *μ*m, line separation of 6 *μ*m, and layer separation of 5 *μ*m. The radial offsets were +2 in all recipients (meaning that all the alignment incisions were outside the trephination) and −2 in all donors (meaning that all the alignment incisions were inside the trephination).

On the anterior side cuts, the spot separation and the layer separation were 3 *μ*m; in the ring lamellar cut (spiral pattern), the tangential spot separation was 5 *μ*m and the radial spot separation was 4 *μ*m; on the posterior side cut, the spot separation was 3 *μ*m and the layer separation was 2 *μ*m. The depth of the lamellar cut of the donor and recipient was 2/3 of the mean corneal thickness of the graft and recipient's eye, respectively. All diameters (anterior side cut, lamellar cut, and posterior side cut) were performed, 0.1 mm larger than the resulting diameter, thus overlapping each other. The donor cornea was placed into an artificial anterior chamber type Barron (Katena, Denville, USA) to achieve trephination from the epithelial side. Each laser procedure requires a disposable glass interface, which applanates the cornea completely during the laser procedure.

**Figure 3:**
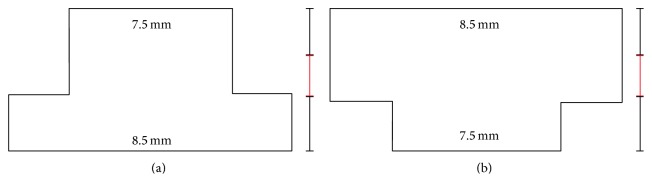
Different profiles of FLAK, upper and lower diameter are given in millimeters. Black and red bars indicate mean corneal thickness divided by 3 (meaning that the cornea was divided in anterior 2/3 and posterior 1/3). (a) Top hat profile used in Fuchs dystrophy enables a higher transplantation rate of corneal endothelial cells. (b) Mushroom profile used in keratoconus patients leads to less endothelial cell transplantation.

For laser trephination of the recipient's cornea, the eye was fixated by means of a vacuum suction ring. The glass cone interface was placed within the suction ring so that the cornea was completely applanated. We performed a complete penetrating laser trephination after which the corneal button was removed with forceps and a spatula under microscopic control. If necessary, a microscissor was used to complete the incision. The top hat profile was used in Fuchs dystrophy, whereas the mushroom profile was used in keratoconus patients (Figure 3).

### 2.5. Suturing

**Figure 4:**
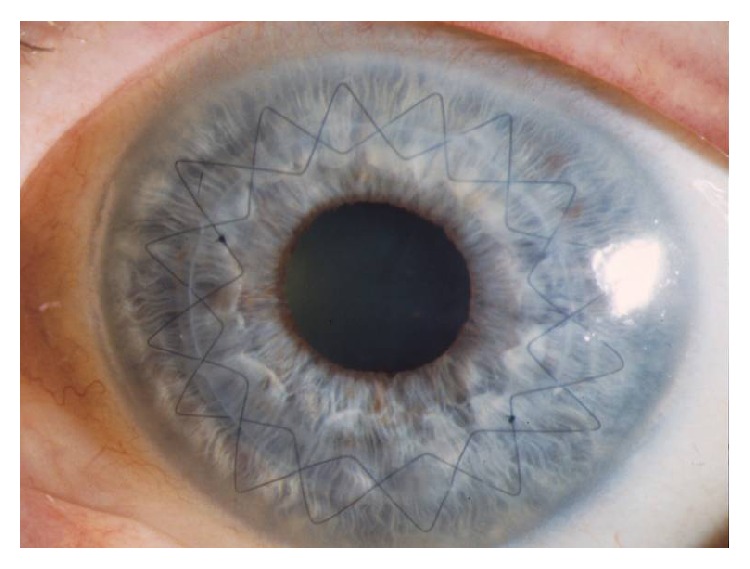
Corneal button sutured with double-running cross-stitch suture six weeks after excimer laser keratoplasty and 8 cardinal sutures for temporary fixation have been removed at the end of surgery.

**Figure 5:**
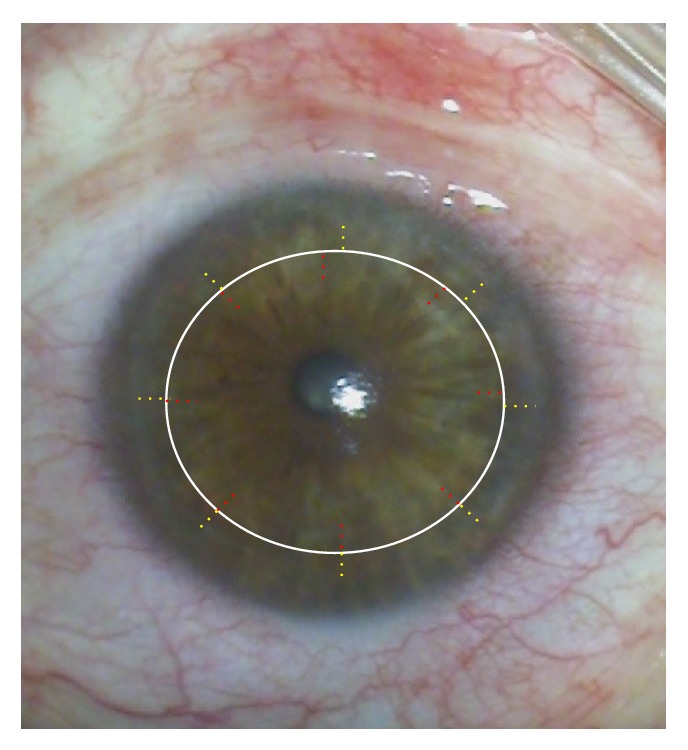
(Hypothetical diagram) different locations of radial incisions in donor and recipient (for better visualization, the red markings are in the donor and the yellow in recipient) after femtosecond laser trephination in keratoconus.

In all patients, a peripheral iridotomy was performed at the 12-o'clock position [16]. After temporary fixation of the donor button in the recipient bed with 8 interrupted sutures, a permanent wound closure was achieved by a 16-bite double-running diagonal cross-stitch suture (10–0 nylon) according to Hoffmann [17] (Figure 4). We attempted to suture as deep as 90% of the total corneal thickness. The eight cardinal sutures were placed at the site of orientation teeth with the excimer laser and at the site of the alignment incisions with the femtosecond laser as well as possible (Figure 5). In cases of wound gaping or graft override, additional interrupted sutures were used to ensure proper donor-host alignment at the end of surgery.

## 3. Results

**Figure 6:**
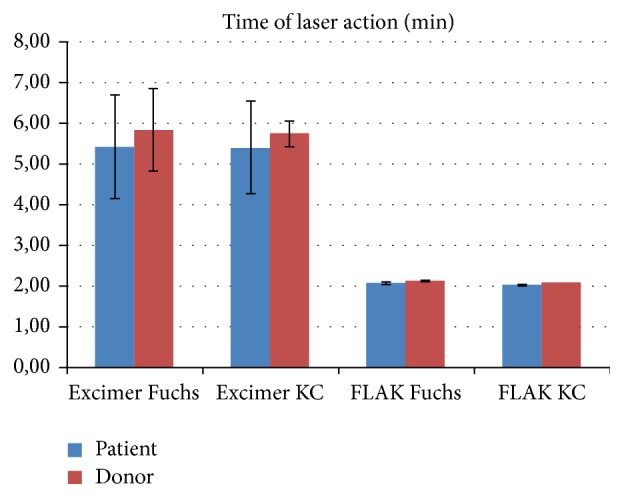
Time of laser action in minutes for all study groups separately for both patient and donor trephinations (Fuchs = Fuchs dystrophy, KC = keratoconus).

**Figure 7:**
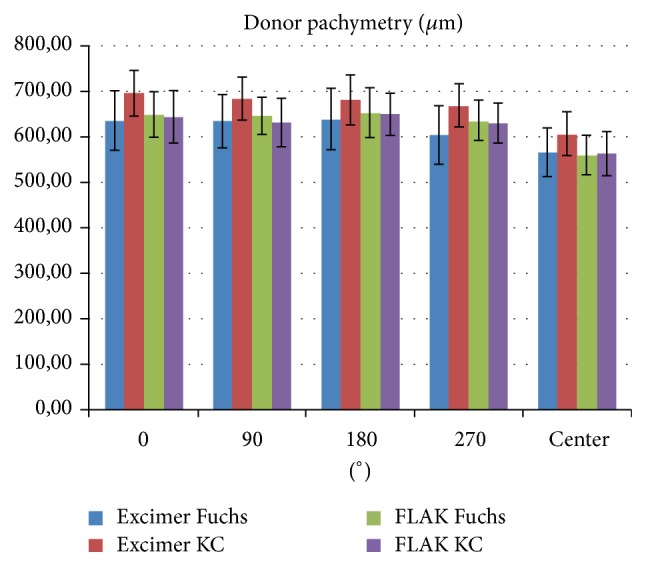
Distribution of pachymetry values for all study groups measured manually with ultrasound pachymetry at the center and in 4 midperipheral points at 0°, 90°, 180°, and 270° (KC = keratoconus, Fuchs = Fuchs endothelial dystrophy).

**Figure 8:**
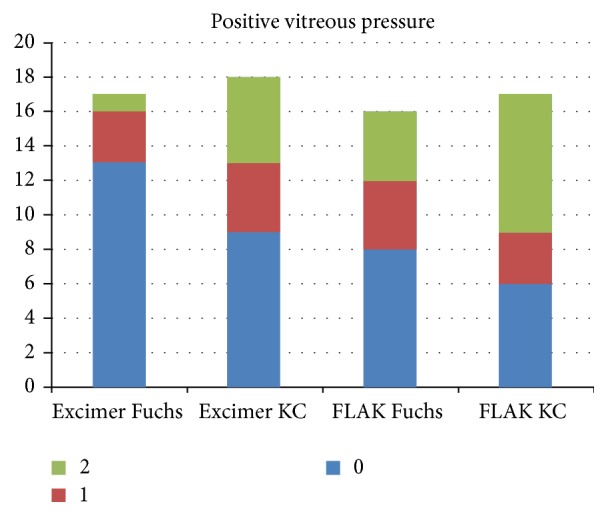
Positive pressure from vitreous (vis a tergo), depicted in three grades: 0 means no intraoperative pressure, 1 means iris prolapse till the level of the corneal incision, and 2 means iris prolapse beyond the level of the corneal incision.

**Figure 9:**
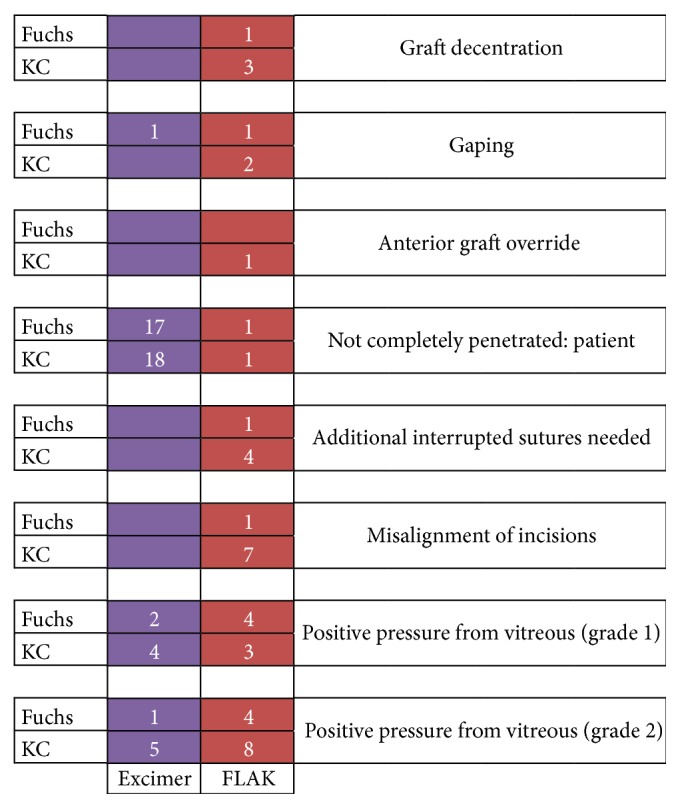
Comparison of excimer versus femtosecond laser-assisted penetrating keratoplasty (FLAK): intraoperative results.

Generally, the laser action time for trephination was much shorter for femtosecond compared to excimer laser trephination (Figure 6). The distribution of pachymetry values for the grafts is depicted in Figure 7. No intraoperative complications have been noticed. Incisions had to be completed with scissors in almost all eyes of groups I/II but only 2 cases in groups III/IV. Decentration happened in none of the eyes in groups I/II, but in 3/1 eyes in groups III/IV. No additional interrupted sutures were necessary for groups I/II, but in 4/1 cases in groups III/IV. Orientation markers were aligned in all cases of the excimer groups; in contrast, orientation markers were not totally aligned in 7/1 cases in groups III/IV. After removal of 8 cardinal sutures, graft override appeared in none of groups I/II/IV but in one case of group III. Moreover, gaping occurred in 0/1 eyes of groups I/II but in 2/1 cases in groups III/IV. Intraoperative positive pressure from vitreous has occurred in all groups as follows: 3/9 eyes in groups I/II and 8/11 in groups III/IV (Figure 8). In particular, positive vitreous pressure grade 2 appeared in 8 patients of the keratoconus FLAK group, but only in 1 patient of the Fuchs excimer laser group. An overview of the above given results is displaced in Figure 9.

## 4. Discussion

Studies comparing an established corneal transplantation procedure with the new femtosecond laser technology that was introduced to clinical practice in 2006 were already carried out [18, 19]. In a recent publication, Birnbaum et al. have compared the results of 123 FLAK with conventional PKP in a randomized clinical study [18]. It has been revealed that despite the potential advantages of the femtosecond procedure it did not provide superior refractive results as compared to mechanical trephination. They found a topographic astigmatism after suture removal of about 6 diopters in the FLAK group.

A major advantage of femtosecond laser is the possibility to create different 3D profiles [18] (with the most widely spread ones being the top hat, mushroom, and zig-zag profile [13, 18]). It is considered mechanically stable [18]. Its stability is derived from the overlap, which is created by the side cut especially in the top hat configuration [20, 21]. Nevertheless, we found in our study that it was difficult to get it watertight without steps and gaps in comparison to the excimer laser keratoplasty. To avoid complications recorded in earlier studies using the mushroom profile (e.g., infiltrates, steps, and ointment deposits), we successfully used an anterior part of the mushroom as thick as two-thirds of the mean of midperipheral donor and recipient thickness. This procedure may be recommended from our point of view.

The fact that FLAK created more gaps after the removal of the cardinal sutures resulted in the necessity to use more single interrupted sutures for correct donor-host adaptation. This was not due to a learning curve of the surgeon because a different cut profile was used in the FLAK group. Although, the mushroom profile has a relatively large diameter at the corneal surface, the wound apposition was less accurate. We have to admit that a suture depth of 90% can only be intended and depends strongly on the experience of the surgeon. We further have to admit that there are no ideal geometrical settings for the mushroom profile. But a more likely explanation is that in keratoconus eyes the applanation done to the cone-shaped bulging cornea results in flattening. We believe that this extreme flattening effect leads to an alteration in the theoretically planned right angles of the anterior, posterior, and lamellar side cuts. Therefore, we got instead of right angles oblique angles and instead of a round trephination an oval- or pear-shaped trephination, which led to difficulty in fitting the properly cut donor button into the somehow distorted recipient bed. In particular, in keratoconus, orientation lines of donor and recipient tended not to match exactly (Figure 5). In such FLAK cases, an interrupted suturing technique might be more appropriate than a double-running suture.

It was obvious that the excimer laser needs more time to penetrate the cornea than the femtosecond laser. This is because the excimer laser digs from the surface a trench into the cornea while its energy is gradually absorbed [1]. On the other hand, the femtosecond laser creates cavitation bubbles at different depths of the cornea and thus an incision can be obtained faster [22]. We intended a complete perforation of the cornea during FLAK. If the femtosecond laser theatre is separated from the surgery room, it might be preferable to leave a stromal gap between 50 and 80 *µ*m and to complete perforation in the surgery room. FLAK achieved an overwhelming number of cases with complete perforation of the cornea in comparison to the excimer PKP.

Another factor that must be considered here is the suturing technique. The double continuous running suture technique remains to be the suture of choice in FLAK [18]. In our study, the suturing was done down deep to the pre-Descemet's layer and not just at the level of the side cut of the profiled graft. In an ordinary PKP or Exc PKP, the suture is placed in the pre-Descemet's region of the donor cornea [23]. In Germany, the double-running cross-stitch suture according to Hoffmann is preferred over interrupted sutures, because it results in earlier visual rehabilitation and higher regularity of topography as long as the sutures are in place and a lower risk of suture loosening and need of suture replacement [17]. In case of corneal thinning, such as that in keratoconus, the suture may run through the anterior chamber at the recipient site.

One of the disadvantages of FLAK is that it generates a higher intraocular pressure than the Exc PKP. This was proved by direct intravitreal measurements [22, 24]. The sclera is inexpansible. Therefore, an increase in the volume of choroidal blood vessels produces disproportionate changes of intraocular volume and thus intraocular pressure. Moreover, it leads to major changes in the osmolarity of the vitreous. When the cornea is removed, the mechanical barrier to vitreous expansion is lost. In this situation, the iris-lens diaphragm is pushed forward (the so-called positive vitreous pressure or “vis a tergo”). In severe cases, it is impossible to maintain the anterior chamber and the cardinal sutures are difficult to place. Even the iris can be sutured to the corneal button thus leading to further difficulties. Moreover, a sudden increase in the intraocular pressure to values which are higher than the perfusion of the retinal vessels was recorded. However, this effect is only for a short time, due to the fast perforation of the cornea. Up to now, no central artery occlusion due to FLAK has been reported. By leaving a stromal gap and finishing the perforation in the surgery room, the suction ring can be removed earlier. This leads to reduction of pressure on the eye, thus enabling intraocular pressure reaching equilibrium and reducing positive vitreous pressure. Moreover, there are now also faster femtosecond laser platforms available which may help to further minimize this risk.

Decentration of corneal grafts in keratoconus patients with FLAK was more frequent than that in patients that were treated with excimer laser. Because of the flat applanation with the FLAK, it was more likely to obtain a decentered oval-shaped recipient incision. In contrast, in the excimer laser PKP, we have been using masks with orientation teeth which allow us to suture the first eight cardinal sutures with small risk of horizontal torsion because, according to the key-keyhole-principle, the orientation teeth in the donor fit exactly into the orientation notches of the recipient. Such a precise orientation is absent in the FLAK, since only radial markings are present. It became obvious that in the femtosecond laser trephined KC eyes these radial incision lines did not fit completely comparing donor and recipient. Therefore, the hypothesis that a better graft alignment can be achieved with FLAK cannot be confirmed with the geometrical settings in our study.

## 5. Conclusion

Intraoperative decentration, misalignment of the donor in the recipient bed, and need for additional interrupted sutures, as well as positive pressure from the vitreous, were more frequent when FLAK was performed.

Future comparative studies with a faster excimer laser platform and a concave femtosecond laser patient interface or even a “liquid interface” are needed to be carried out. These studies might show us less intraoperative complications regarding both techniques. The next step of our group is the presentation of best-spectacle corrected visual acuity, postoperative astigmatism, and regularity of topography after removal of all sutures in all eyes.
